# Automated Injectors versus Manual Administration: A Comparative Analysis of Radiation Exposure Reduction in ^18^ F-FDG Delivery

**DOI:** 10.1055/s-0044-1791781

**Published:** 2024-10-16

**Authors:** Saumya Srivastava, Subhash Chand Kheruka, Sanjay Gambhir, Pankaj Tandon, Anjali Jain, Sumit Kumar, Naema Al-Maymani, Khulood Al-Riyami, Rashid Al-Sukaiti

**Affiliations:** 1 Department of Nuclear Medicine, Sanjay Gandhi Postgraduate Institute of Medical Sciences, Lucknow, Uttar Pradesh, India; 2 Department of Radiology & Nuclear Medicine, Sultan Qaboos Comprehensive Cancer Care, and Research Center, Muscat, Oman; 3 Division of Radiological Safety, Atomic Energy Regulatory Board, Mumbai, Maharashtra, India; 4 Department of Health Research Multidisciplinary Research Unit, KGMU, Lucknow, Uttar Pradesh, India

**Keywords:** ^18^ F-FDG, autoinjector, IRIS, TLD, manual injection

## Abstract

**Background** Despite the presence of safety protocols, the manual manipulation of radiopharmaceuticals continues to pose a significant occupational radiation risk. Health care professionals in nuclear medicine are at risk of radiation exposure, particularly to their hands and eyes. Despite existing protective measures, manual handling of radiopharmaceuticals remains a significant source of occupational radiation.

**Objective** This study evaluates the effectiveness of automated injectors in reducing radiation exposure among health care workers during fluorine-18 fluorodeoxyglucose ( ^18^ F-FDG) administrations, compared with traditional manual injection methods.

**Methods** We assessed radiation exposure levels associated with manual versus automated ^18^ F-FDG injection techniques using specialized dosimeters. Measurements focused on whole-body, extremity, and eye-lens radiation doses to evaluate the potential benefits of automation in minimizing exposure.

**Results** Findings reveal that automated injectors significantly reduce radiation exposure, with decreases of 97.97 and 98.96% in left- and right-hand extremity doses, respectively, 43.24% in eye-lens dose, and 91.66% in whole-body dose compared with manual methods.

**Conclusion** Automated injection systems offer considerable advantages in reducing health care worker radiation exposure in nuclear medicine. The substantial reduction in staff doses underscores the necessity of transitioning to such technology to promote safer clinical environments. This study highlights the critical role of automation in enhancing occupational safety standards within diagnostic radiology settings.

## Introduction

Nuclear medicine and radiation protection specialists are dedicated to reducing radiation exposure for patients, staff, and anyone involved in medical procedures. With the increasing use of positron emission tomography (PET) imaging, particularly employing fluorine-18 fluorodeoxyglucose ( ^18^ F-FDG), concerns over radiation hazards in both medical and occupational settings have become more pronounced. 1 2 3 PET imaging involves the intravenous injection of radiopharmaceuticals like ^18^ F-FDG, necessitating strict adherence to guidelines that protect against radioactive and microbiological hazards.

A variety of preventative measures are employed to shield operators from radiation exposure. These include maintaining distance from radiation sources, utilizing radiation syringe shields, and using materials that attenuate radiation intensity. For example, using lead containers for transporting ^18^ F-FDG and enclosing radiation sources with absorbent materials perhaps can lower the contamination levels. Such precautions are vital in nuclear medicine facilities to ensure compliance with radiation safety regulations.

Despite these protective measures, manual administration of ^18^ F-FDG remains a significant source of occupational radiation exposure for nuclear medicine technicians. PET procedures are known to result in higher whole-body and extremity doses than single-photon emission computed tomography (SPECT) procedures, with particular risks to fingers and eyes. 4 5 6 7 8 9 Consequently, there is a critical need to optimize radiation doses to keep staff exposure within safe limits.

In response to these concerns, shielded automated infusion systems have been introduced to further mitigate staff exposure to radiation. The past decade has seen a surge in the use of PET in diagnostic nuclear medicine, driven by advancements in PET/computed tomography (CT) technology. Although these advancements have reduced radiation hazards to patients, health care workers continue to face exposure risks, especially during manual injections. This underscores the importance of employing optimization measures such as automated injectors to minimize radiation doses. 7 8

Despite advancements in technology, health care workers are still at risk of notable radiation exposure, which, in some cases, exceeds recommended occupational dose limits. Automated systems, like the Posijet from Lemer Pax, France, offer a solution to minimize this risk, ensuring a safer working environment and compliance with regulatory guidelines.

Given the dual aims identified within the introduction, it is essential to consolidate these into a singular, overarching goal. Therefore, the primary objective of this study is to assess and compare the radiation doses to health care workers associated with manual versus automated injection techniques in the administration of ^18^ F-labeled radiopharmaceuticals. This includes evaluating worker exposure before and after the implementation of an automated system, to delineate the benefits such systems offer in reducing radiation exposure during the administration process.

## Material and Methods

This was a study performed in a very busy institute to analyze the radiation exposure of occupational workers involved in administering ^18^ F-FDG to patients.

### Radiation Exposure Measurement

**Fig. 1:**
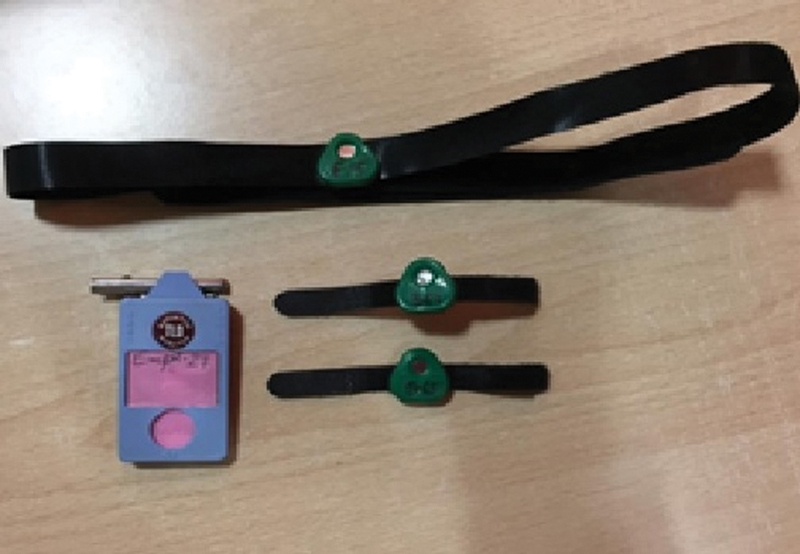
Chest, extremities, and eye dosimeters.

The radiation exposure was measured with the help of thermoluminescent dosimeters (TLDs) embedded in personnel monitoring badges; these dosimeters consisted of calcium sulfate doped with dysprosium (CaSO _4_ :Dy). Specific doses were measured using various forms of badges: a chest badge for whole-body dose, a ring badge for extremity (finger) dose, and a head badge for eye dose ( Fig. 1 ).

### Patient Cohort and Dosage Administration

**Fig. 2:**
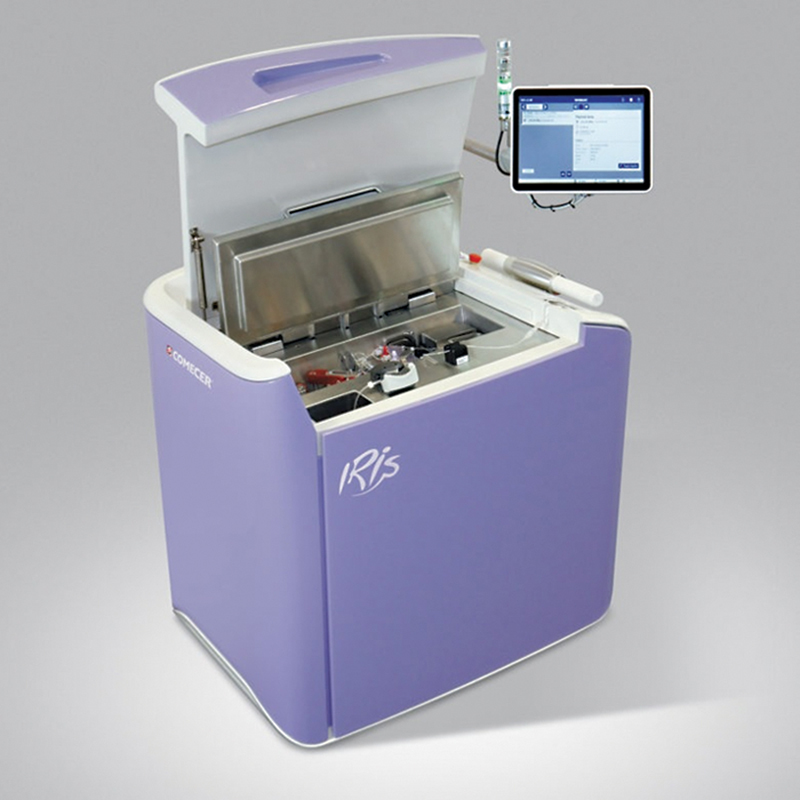
The IRIS autoinjector.

A total of 99 patients participated in the study. Of these, 55 were administered approximately 370 MBq of ^18^ F-FDG injected manually, as per the literature in Table 1 . The other 44 patients received the same quantity through an automated injector (IRIS) manufactured by COMECER, Italy, as illustrated in Table 2 . Fig. 2 illustrates the automated injector used in the present study.

**Table 1:** TLDs (chest, ring, and head badges) and FDG doses used for manual injection process

Sl. no.	Date	Time taken per injection (s)	Injected dose (MBq)
1	February 15, 2019	60	357.79
2	February 15, 2019	60	349.28
3	February 15, 2019	60	389.61
4	February 18, 2019	90	389.98
5	February 18, 2019	90	368.52
6	February 18, 2019	90	404.04
7	February 20, 2019	110	390.35
8	February 20, 2019	60	199.80
9	February 20, 2019	90	479.15
10	February 20, 2019	75	370
11	February 20, 2019	90	378.14
12	February 20, 2019	60	450.66
13	February 21, 2019	90	303.40
14	February 21, 2019	60	447.70
15	March 5, 2019	60	445.48
16	March 5, 2019	60	388.87
17	March 5, 2019	60	410.07
18	March 5, 2019	75	452.88
19	March 5, 2019	90	366.30
20	March 6, 2019	60	404.04
21	March 6, 2019	60	368.52
22	March 6, 2019	60	485.07
23	March 6, 2019	120	388.50
24	March 6, 2019	90	366.30
25	March 6, 2019	60	372.59
26	March 6, 2019	75	358.90
27	March 8, 2019	75	361.86
28	March 8, 2019	60	339.29
29	March 8, 2019	60	379.25
30	March 8, 2019	60	416.25
31	March 8, 2019	90	399.60
32	March 8, 2019	90	373.70
33	March 8, 2019	65	344.10
34	March 8, 2019	75	333.00
35	March 8, 2019	63	373.70
36	March 11, 2019	75	435.86
37	March 11, 2019	66	395.90
38	March 11, 2019	75	336.70
39	March 11, 2019	80	362.60
40	March 11, 2019	80	395.90
41	March 11, 2019	70	347.80
42	March 12, 2019	50	405.89
43	March 12, 2019	60	404.04
44	March 12, 2019	80	391.46
45	March 12, 2019	60	394.42
46	March 12, 2019	75	351.50
47	March 12, 2019	65	370.00
48	March 12, 2019	75	355.57
49	March 12, 2019	60	381.10
50	March 13, 2019	60	407.00
51	March 13, 2019	55	381.10
52	March 13, 2019	70	377.40
53	March 15, 2019	60	352.98
54	March 15, 2019	60	297.48
55	March 15, 2019	75	330.78

Abbreviations: FDG, fluorodeoxyglucose; TLD, thermoluminescent dosimeter.

**Table 2:** TLDs (chest, ring, and head badges) and FDG doses used for automated injection process

Sl. no.	Date	Time taken per injection (s)	Injected dose (MBq)
1	February 4, 2019	180	364.08
2	February 4, 2019	180	368.89
3	February 5, 2019	180	379.62
4	February 5, 2019	180	220.89
5	February 6, 2019	180	382.58
6	February 6, 2019	180	381.47
7	February 7, 2019	180	365.56
8	February 7, 2019	180	368.52
9	February 7, 2019	180	368.89
10	February 8, 2019	180	366.67
11	February 8, 2019	180	371.85
12	February 11, 2019	180	371.85
13	February 11, 2019	180	379.62
14	February 12, 2019	180	380.36
15	February 12, 2019	180	233.10
16	February 14, 2019	180	375.18
17	February 14, 2019	180	366.67
18	February 18, 2019	180	370.37
19	February 18, 2019	180	361.86
20	February 21, 2019	180	365.93
21	February 21, 2019	180	356.68
22	February 21, 2019	180	368.52
23	February 21, 2019	180	374.44
24	February 21, 2019	180	369.26
25	February 25, 2019	180	375.55
26	February 25, 2019	180	376.66
27	February 25, 2019	180	368.52
28	February 25, 2019	180	366.67
29	February 25, 2019	180	370.00
30	February 26, 2019	180	364.82
31	February 26, 2019	180	363.71
32	February 26, 2019	180	349.28
33	March 1, 2019	180	368.89
34	March 1, 2019	180	371.11
36	March 1, 2019	180	372.96
37	March 1, 2019	180	527.25
38	March 1, 2019	180	367.41
39	March 1, 2019	180	365.19
40	March 6, 2019	180	369.26
41	March 6, 2019	180	365.19
42	March 7, 2019	180	203.50
43	March 8, 2019	180	358.90
44	March 8, 2019	180	370.00

Abbreviations: FDG, fluorodeoxyglucose; TLD, thermoluminescent dosimeter.

### Data Collection and Reader Models

**Fig. 3:**
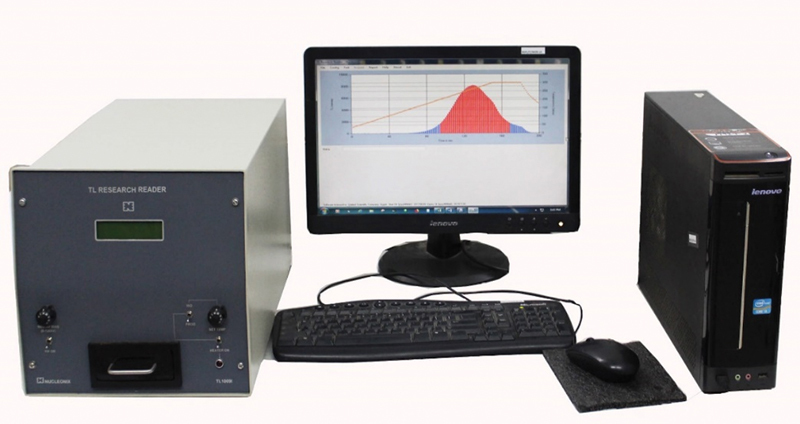
Thermoluminescent dosimeter (TLD) badge reader for the chest.

**Fig. 4:**
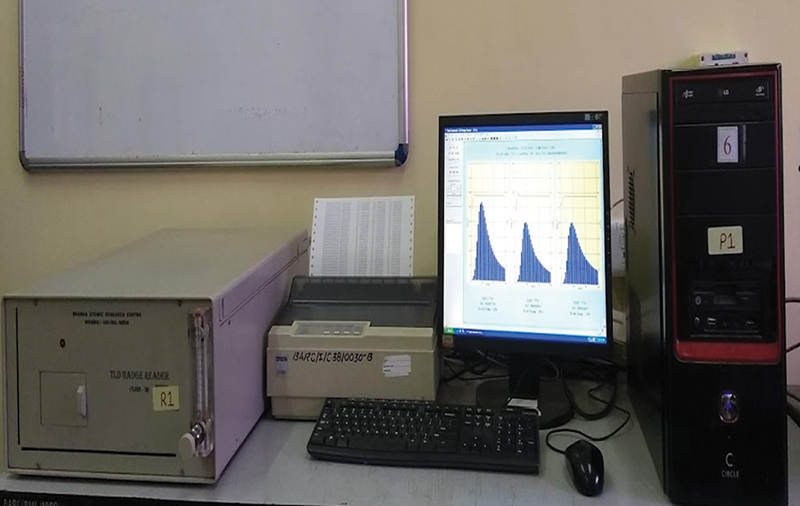
Ring and head thermoluminescent dosimeter (TLD) badge reader along with a personal computer (PC) and other accessories.

Radiation exposure data were collected with instruments inherently designed for this purpose called readers. Ring badges and eye lens were read using the Nucleonix TL Research Reader (Type TL 1009I) with a personal computer ( Fig. 3 ). Chest TLD badges were also read using a TLD badge reader made by BARC (Model BR 7B; Fig. 4 ).

### Cross-Calibration Process

A very detailed cross-calibration process was performed to make responses of different TLD reader models consistent and accurate. This consisted of the following:

- Cross-calibration: Every make of TLD reader was cross-calibrated to a reference standard by reading the same package of dosimeters with known radiation doses, and calibration factors were corrected accordingly.
- Consistent material for dosimeters: All dosimeters made use of calcium sulfate doped with dysprosium (CaSO4:Dy), which resulted in uniform response among the different kinds of badges.
- Standardized calibration factors: To reduce variations in the measurements of doses, it was necessary to standardize calibration factors for use with each model of readers.
- Control of external variables: The background radiation level, humidity, and handling of dosimeters were all monitored through the study to give accurate readings.

All these measures ensured proper data without any bias or interference from whichever type of reader was used.

### Frequency of Measurement

Measurements were collected constantly during the ^18^ F-FDG injection administrations; hence, no time interval or postinjection period was set for measurements. Such constant monitoring provides much more accurate and relevant information regarding the doses of radiation being reported to the personnel.

### Other Considerations

Background readings, moisture, and dosimeter handling are factors that this study will control as they may have an influence on overall reliability and control an effect of differing TLD reader systems.

### Dose Assessment Algorithm

An algorithm has been developed and validated for processing dosimeter readings to obtain radiation dose values for extremity and eye-lens dosimeters. The major salient features are as follows:

- The general view of the algorithm is it processes TLD data to uncorrected readings in a set of radiation dose values taking into account the type and energy of the radiation measured.
- The performance of the algorithm was validated through extensive dosimeter testing exposed to known radiation doses in controlled conditions. The results were compared with those obtained through standard methods of dose evaluation to ensure consistency.
- Uniformity and reliability: The algorithm furnishes uniform and reliable dose estimates over different types of dosimeters and changing radiation conditions.

#### Manual Injection

**Fig. 5:**
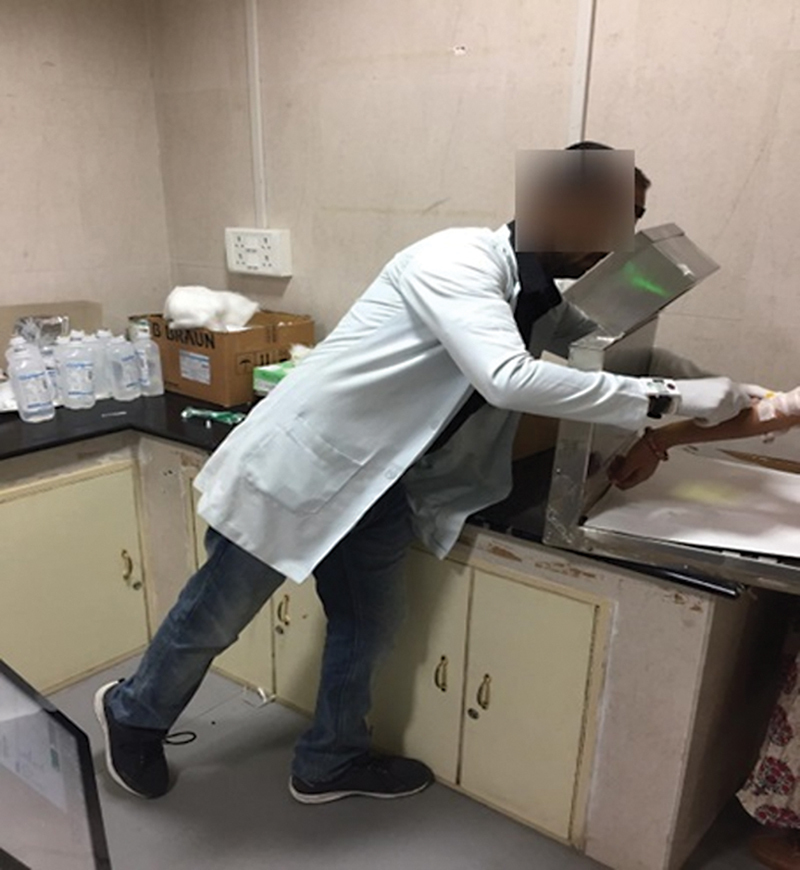
Manual injection process.

Patients are fitted with an intravenous cannula in their veins before injection to minimize injection time. A prepared dose of ^18^ F-FDG in a syringe is measured using a dose calibrator (ionization chamber, BIODEX, ATOMLABTM 500) and then administered to the patients. The entire process takes place behind a lead shield (L-Bench with 16.6-mm lead shielding), as shown in Fig. 5 . Before being administered to the patient, the syringes are placed inside a syringe shield for added safety. To transport the syringe loaded with ^18^ F-FDG, a lead syringe holder is utilized.

During the study, a total of six sets of dosimeter badges were utilized to monitor the radiation exposure levels for the manual injection protocol of ^18^ F-FDG. Each set of badges consisted of one whole-body dosimeter, one eye dosimeter, and two ring badges (left and right marked on them) to assess radiation exposure on the extremities.

For the automatic injection process, two sets of badges were used, with each set worn by a different person involved in administering the ^18^ F-FDG doses using the automated injector. This setup allowed for the evaluation of radiation exposure for personnel using the automated system.

The manual injection process required rotation between personnel to administer the doses. Therefore, four sets of badges were used by four different persons who took turns performing manual injections. This approach ensured comprehensive data collection for radiation exposure during manual injections, considering the varying exposure levels based on individual techniques and movements.

By employing these multiple sets of dosimeter badges, the study could accurately measure and compare radiation exposure among personnel involved in both the manual and automated injection processes. This comprehensive approach provided valuable data to evaluate the safety and efficacy of the automated dispenser and injector system in minimizing occupational radiation doses for health care workers in nuclear medicine facilities.

#### Automated Injection

An autoinjector machine (COMECER INJECTOR – IRIS) was utilized, as depicted in Fig. 1 . To ensure a leakage-free injection process, a sterile cassette set was connected to the autoinjector after thorough valve checks. Inside the injector, a waste vial was positioned, and a 500-mL sterile saline solution was connected to the injection set. In preparation for ^18^ F-FDG administration and to minimize injection time, patients had an intravenous cannula fitted in their veins. The cannula was connected to the injector cassette set using patient line tubing for the administration of the measured activity (in MBq/mL). Before administering the radiotracer (i.e., ^18^ F-FDG), the vein was checked for any obstructions by flushing 2 mL of 0.9% NaCl. Subsequently, only the radiotracer substance was administered.

During the automated administration of ^18^ F-FDG, occupational workers were instructed to maintain a safe distance from the administration point to minimize radiation hazards. The tubing connected to the patient was disposed of after a single use. The administered dose and injection time were recorded in a log book.

**Fig. 6:**
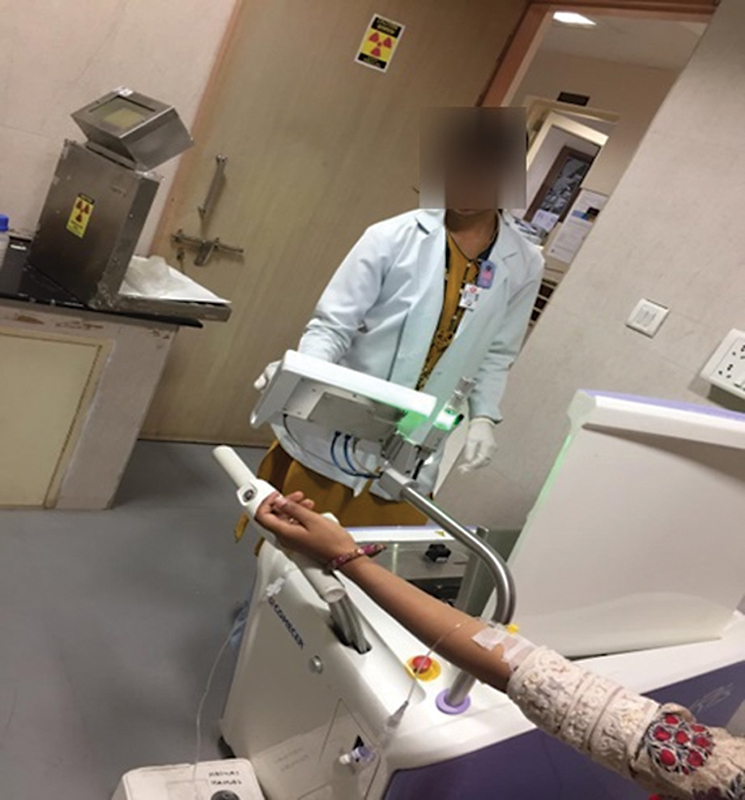
Automatic injection process of fluorine-18 fluorodeoxyglucose ( ^18^ F-FDG) using the IRIS autoinjector.

Fig. 6 provides an overview of all the operator functions of the automated injector, while Table 2 details the TLDs and the various FDG doses used for the automated injection process.

### Extremity (Finger) and Eye-Lens Badges

- The measurement of extremity (finger) dose and eye-lens dose was performed using a ring badge and an eye-lens dosimeter (head badge). Both badges are composed of three CaSO4:Dy Teflon disks with dimensions of 0.4-mm thickness and 5.0-mm diameter.
- Dosages were assessed using an algorithm specifically developed for these dosimeters. This algorithm is integral to the study methodology. It processes data from the TLDs, converting readings into radiation dose values for extremity (finger) and eye-lens dosimeters. This tailored algorithm ensures accurate and consistent interpretation of dosimetry data. It is pivotal in accurately quantifying the radiation doses received by the extremities and eye lenses of health care workers during the administration of ^18^ F-FDG, thereby enhancing the reliability and precision of the study's findings.
- For this experimental exercise, the reporting dosage for these dosimeters is set at 0.5 mSv, and any evaluated dose below 0.5 mSv is recorded as 0.0 mSv.

### Whole-Body Dosage Using Chest Badge

- The whole-body dosage values were measured by wearing a TLD badge at chest level.
- The doses were evaluated using the standard dose evaluation algorithm typically employed in TLD personal monitoring services.
- The radiation type (X-rays, beta, gamma) and energy were identified based on the reading pattern and response of the three disks in the TLD badge.

### Features of used TLD Badge

The Bhabha Atomic Research Centre (BARC) has innovatively designed and developed a new compact three-element extremity ring badge (ERB) dosimeter. This ERB dosimeter was employed specifically to measure radiation doses to the whole body, eyes, and fingers of the operators. To achieve this, TLDs were utilized, which were the following:

- Based on the indigenously developed CaSO _4_ :Dy Teflon disks, prepared in a proportion of 1:3 with a 0.05 mole % Dy content. Disk specifications are shown in Table 3 .

**Table 3:** Specifications for three different types of disks, detailing their material composition, thickness, diameter, physical thickness, and weight

Disk	Material	Thickness (mg/cm ^2^ )	Diameter (mm)	Thickness (mm)	Weight (mg)
Disk 1	Mylar	7	5.0	0.4	20
Disk 2	Copper and Perspex	1,000	5.0	0.4	20
Disk 3	Teflon	300	5.0	0.4	20

Notable features of the ERB dosimeter include the following:

Ability to cover a broad dose range from 0.5 mSv to 10 Sv.

Proficiency in discriminating the type and energy of radiation, namely beta, gamma, and low-energy X-rays (up to 200 keV).

Capability to provide measurements of equivalent doses in terms of the operational quantity Hp(0.07).

This advanced dosimeter from BARC marks a significant leap in ensuring precision and safety in radiation measurements, safeguarding health care professionals and researchers alike.

## Results

The average radioactivity of ^18^ F-FDG dispensed by the COMECER INJECTOR – IRIS was found to be 379.62 MBq (range: 203.5–527.25 MBq), whereas, with the manual technique, it was 362.23 MBq (range: 297.5–485.1 MBq).

When using the IRIS system, the sums of the whole-body dose, eye dose, and finger dose (right and left) per the PET procedure for individual operators were significantly lower at 91.66, 43.24, and 98.96 and 97.97%, respectively, compared with the manual injection technique for ^18^ F-FDG.

In the assessment of extremity doses received during ^18^ F-FDG handling and injection into the patient, a TLD ring dosimeter was employed. The total radioactivity of ^18^ F-FDG handled by operators using the IRIS system and manual injection technique was 15,587.73 and 20,868.00 MBq, respectively.

During the manual injection process, radiation exposure rates for different parts of the body were as follows:

- **Finger dose (right and left):** The exposure rates were 0.185 and 0.212 μSv/MBq, translating to total doses of 3.87 and 4.44 mSv, respectively.
- **Eye-lens dose:** The exposure rate was 0.017 μSv/MBq, amounting to a total dose of 0.37 mSv.
- **Whole-body dose:** The exposure rate was 0.0057 μSv/MBq, resulting in a total dose of 0.12 mSv.

When using the IRIS system, a significant reduction in radiation exposure was observed:

- **Finger dose (right and left):** The exposure rates were notably reduced to 0.00577 and 0.0025 μSv/MBq, leading to total doses of 0.09 and 0.04 mSv, respectively.
- **Eye-lens dose:** The exposure rate using the IRIS system was 0.0134 μSv/MBq, totaling a dose of 0.21 mSv.
- **Whole-body dose:** The exposure rate was remarkably lower at 0.00064 μSv/MBq, yielding a minimal dose of 0.01 mSv.

**Fig. 7:**
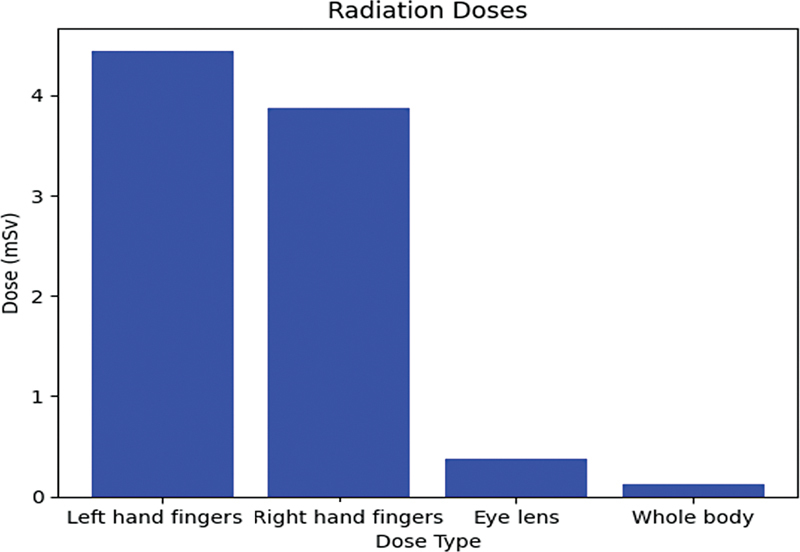
Received calculated dose from the manual injection process.

**Fig. 8:**
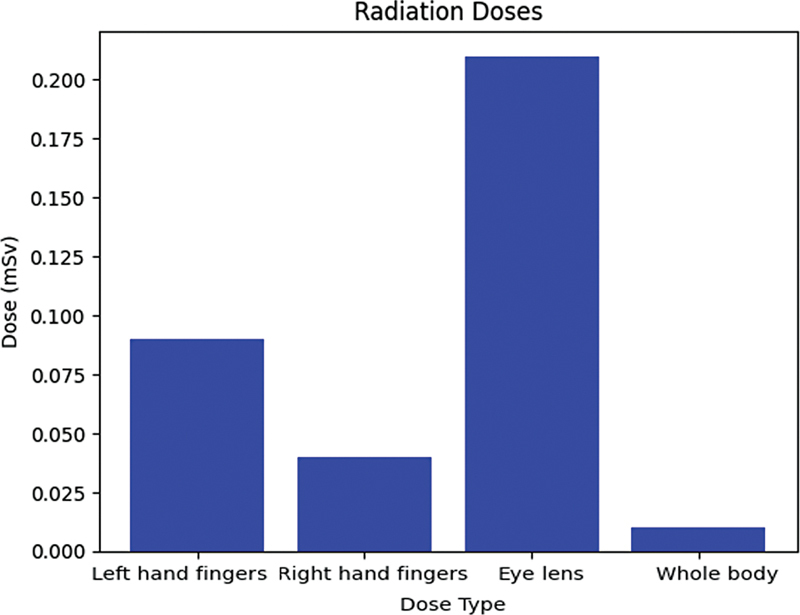
Received calculated dose from the automated injection process.

These variations in radiation exposure between manual injection and the IRIS system are illustrated in Tables 4 and 5 , as well as in Figs. 7 and 8 .

**Table 4:** Individual sum of doses (finger, eye, and whole body) received from different sets of dosimeters during 55 manual injection process

Sl. no.	Received calculated doses during 55 manual injection process	
1	Left hand fingers (mSv)	4.44
2	Right hand fingers (mSv)	3.87
3	Eye lens (mSv)	0.37
4	Whole body (mSv)	0.12

**Table 5:** Individual sum of doses (finger, eye, and whole body) received from different sets of dosimeters during 44 automated injection process

Sl. no.	Received calculated doses during 44 automated injection process	
1	Left hand fingers (mSv)	0.09
2	Right hand fingers (mSv)	0.04
3	Eye lens (mSv)	0.21
4	Whole body (mSv)	0.01

The data highlight the efficacy of the IRIS system in minimizing radiation exposure, especially when contrasted with the manual injection process. Although the eye-lens dose sees a slight increase with the IRIS system, the significant reductions in finger and whole-body doses underscore the system's overall benefit in enhancing radiation safety. This underlines the importance of adopting advanced methods and technologies, like the IRIS system, to mitigate the risks associated with radiation exposure, ensuring a safer working environment for health care professionals in nuclear medicine facilities.

**Fig. 9:**
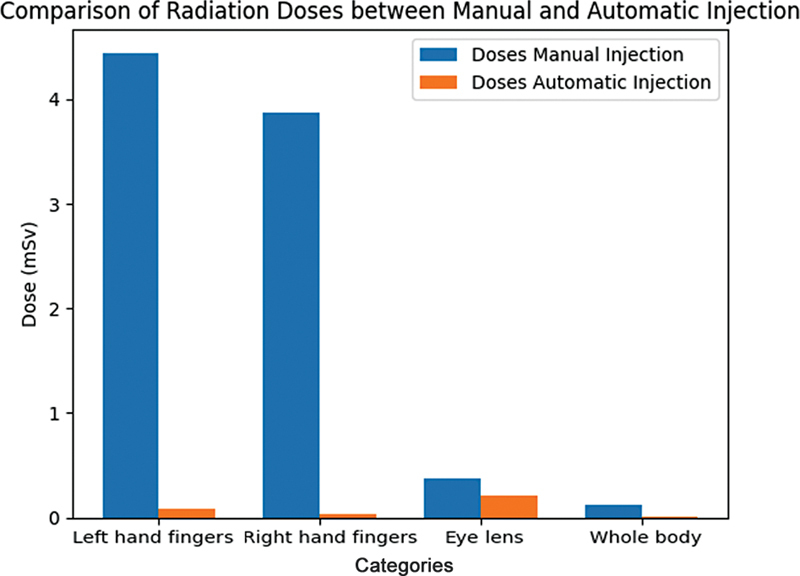
Comparison between calculated dose received from the manual injection and automated injection processes.

**Fig. 10:**
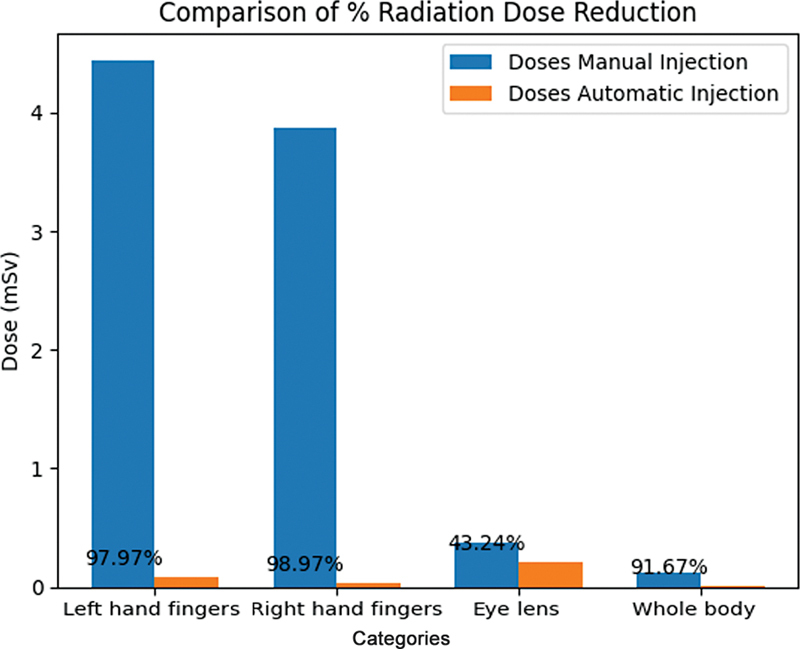
Percentage of radiation dose reduction from manual injection to automated injection process.

The use of the combined dispenser and injector system significantly reduced the operator's fingers dose (right and left), eye-lens dose, and whole-body dose by 98.96, 97.97, 43.24, and 91.66%, respectively, as shown in Figs. 9 and 10 .

## Discussion

This research highlights the significant difficulties faced by technicians and physicians in the field of nuclear medicine as a result of their regular exposure to radiation. To mitigate this susceptibility, the research proposes a comprehensive strategy for safeguarding against radiation, encompassing the strategic implementation of the inverse square law and the utilization of shielding during medical interventions. The inverse square rule has been observed to emphasize the notable influence that augmenting the distance from the radiation source may provide in mitigating exposure.

It is advisable to implement this approach whenever feasible, in addition to direct shielding techniques that establish a tangible barrier between the radiation source and health care personnel. The utilization of automated injectors for the delivery of doses serves to further reduce the time personnel spend near high-dose sources, successfully mitigating occupational exposure.

Furthermore, the research underscores the significance of rigorous compliance with regulatory protocols, specifically those established by the International Commission on Radiological Protection (ICRP) Publication 103 (2007), which promotes the optimization of radiation protection measures to guarantee that radiation doses are maintained at the lowest feasible level (as low as reasonably achievable or ALARA). By employing a combination of shielding techniques, utilizing the principles of the inverse square law, and including automated dispensing systems, these guidelines provide a complete strategy for reducing radiation exposure in nuclear medicine settings.

Through the implementation of these measures, nuclear medicine facilities have the potential to augment the safety and safeguarding of their personnel, thus successfully tackling the obstacles associated with occupational radiation exposure. The comprehensive methodology employed in this approach not only aligns with established protocols and regulatory guidelines but also utilizes principles of physics and technology progress to enhance the operational conditions for health care practitioners engaged in the delivery of radiopharmaceuticals. Monitoring levels of exposure not only guarantees adherence to these requirements but also promotes a more secure work environment. Individuals engaged in the administration of injections and the scanning of patients are more susceptible to radiation exposure within a PET facility. The results suggest that physicians experience elevated radiation exposures, particularly to their hands while administering dosages manually. Potential strategies for mitigating these exposures encompass the implementation of physician rotation by the workload, the use of syringe shields, and adherence to the principles of time, distance, and shielding while handling PET radiopharmaceuticals.

The research emphasizes the benefits of using automated injectors as a means to mitigate radiation exposure.

Our study also references the publication titled “Occupational Radiation Dosimetry Assessment Using an Automated Infusion Device for Positron-Emitting Radiotracers” by A. Robert Schleipman and Victor H. Gerbaudo (2022) 10 , which demonstrates a significant reduction in radiation exposure through the use of automated infusion devices. The results of our study are consistent with this prior research, showing a notable decrease in occupational extremity and whole-body doses during automated FDG administration.

## Conclusion

This study advocates for minimizing radiation exposure in nuclear medicine facilities. By implementing shielding, adhering to guidelines, and utilizing automated injectors, significant reductions in radiation doses can be achieved. Continual monitoring of exposure and adherence to guidelines ensures a safer work environment, promoting the health of the staff and quality patient care.

This study also demonstrates the efficacy and safety of automated dispensing and administration systems in nuclear medicine facilities for minimizing occupational radiation doses. The use of IRIS, a combined dispensing and injection device, was evaluated and was confirmed to be a safe alternative to the conventional manual injection technique. Implementing this automated approach offers a net advantage by significantly reducing radiation doses to personnel.

The study focused on assessing the whole-body, extremities (fingers), and eye-lens doses during the delivery of single doses of ^18^ F-FDG, comparing data from both manual and automated processes. The results revealed a remarkable reduction in radiation exposure when using the combined dispenser and injector system.

Specifically, the study observed a significant decrease in exposure levels of the whole body (–91.66%), extremities (left fingers: –97.97% and right fingers: –98.96%), and eye lens (–43.24%) during the injection phase with the use of the combined dispenser and injector device, compared with manual injection. These results strongly support the effectiveness of the automated system in minimizing radiation exposure for health care workers administering ^18^ F-FDG.

However, it is important to consider some limitations of the study. First, the sample size might have been relatively small, which could potentially limit the generalizability of the findings to a broader population of health care workers in different nuclear medicine facilities. Moreover, the study's duration might have been constrained, and radiation exposure assessments were performed during a specific period, which may not fully capture potential variations in exposure over time.

Additionally, while the automated system shows promise in reducing radiation exposure during the injection phase, the study did not explore potential radiation exposure during other stages of the ^18^ F-FDG administration process, such as the handling and preparation of the radiopharmaceutical. Further investigation into other stages of the procedure could provide a more comprehensive understanding of radiation exposure risks throughout the entire process.

Furthermore, individual variations in health care workers' behavior or movements during the injection procedure, which could impact radiation exposure levels, may not have been fully accounted for in the study. Factors such as operator experience and technique might play a role in radiation exposure, but they were not extensively examined.

Despite these limitations, the study remains a valuable contribution to the field, offering important insights into the benefits of adopting automated injectors to enhance radiation safety in nuclear medicine facilities. Future research with larger sample sizes, longer durations, and consideration of other aspects of the administration process would further strengthen the evidence for the efficacy of automated systems in reducing health care workers' radiation exposure. These findings reinforce the importance of continuous efforts to optimize radiation safety measures for medical personnel working with radiopharmaceuticals.

While IRIS streamlines the process by utilizing the multidose vial for planned doses throughout the day, the physical handling of radioactive vials by a skilled operator in a properly protected environment is still necessary for dispensing radioactive tracers. However, the implementation of the combined dispenser and injector system eliminates the need to transfer radioactive syringes from the radiopharmaceutical laboratory to the injection location, minimizing potential risks. Overall, the study highlights the crucial role of a radiopharmaceutical laboratory in supporting radiation safety practices and ensuring high-quality patient care in the field of nuclear medicine.

In conclusion, this study demonstrates the efficacy and safety of using the automated dispensing and administration system, exemplified by IRIS, to minimize radiation exposure for health care personnel during ^18^ F-FDG PET procedures. The specific conclusions drawn from this study are based on comparing radiation exposure levels between manual and automated injection methods.

The study's results showed a remarkable reduction in radiation exposure levels when using the combined dispenser and injector system. Notably, there was a significant decrease in the exposure levels of the whole-body (–91.66%), extremities (left fingers: –97.97% and right fingers: –98.96%), and eye lens (–43.24%) during the injection phase compared with manual injection.

These findings provide robust evidence supporting the adoption of automated injectors to enhance radiation safety in nuclear medicine facilities. The study's focus on directly comparing radiation exposure levels between manual and automated injection methods adds to the novelty and strength of its conclusions.

While this conclusion aligns with previous studies' findings, the specific contributions of this research lie in its comprehensive evaluation of radiation exposure during ^18^ F-FDG PET procedures and its direct comparison between manual and automated injection methods. The study underscores the significant benefits of adopting automated injectors, such as IRIS, to achieve substantial reductions in radiation doses for occupational workers, creating an optimized and safer environment in nuclear medicine facilities.
